# Therapeutic potential of traditional Chinese medicine in diabetic cardiomyopathy: a review

**DOI:** 10.3389/fendo.2025.1532565

**Published:** 2025-07-23

**Authors:** Wencan Li, Xiang Liu, Zheng Liu, Qichang Xing, Renzhu Liu, Qinxuan Wu, Jiani Zhang

**Affiliations:** ^1^ Department of Clinical Pharmacy, Xiangtan Central Hospital (The Affiliated Hospital of Hunan University), Xiangtan, Hunan, China; ^2^ Hunan Provincial Key Laboratory of the Research and Development of Novel Pharmaceutical Preparations, the “Double-First Class” Application Characteristic Discipline of Hunan Province (Pharmaceutical Science), Changsha Medical University, Changsha, Hunan, China; ^3^ Department of Pharmacy, Xiangtan Central Hospital (The Affiliated Hospital of Hunan University), Xiangtan, Hunan, China

**Keywords:** diabetic cardiomyopathy, traditional Chinese medicine, inflammation, oxidative stress, myocardial fibrosis, autophagy

## Abstract

Diabetic cardiomyopathy (DCM) is a common cardiovascular complication that could lead to changes in cardiac structure and function. It is one of the leading causes of death in diabetic patients. Due to the complex pathogenesis of DCM, there is currently no specific drug or prophylactic agent to treat DCM, so there is an urgent need to identify new therapeutic agents or complementary and alternative therapies for DCM. Although Traditional Chinese medicine (TCM) has some limitations, it has the unique advantages of multi-level, multi-target and few side effects, which could effectively deal with the complex pathological mechanism of DCM. Growing evidence suggests that inflammation, oxidative stress, myocardial fibrosis and autophagy are the main pathological mechanisms of DCM. This study will shed light on the prospects of TCM treatment of DCM from the above perspective in order to provide more ideas and evidence for the clinical use of TCM for the prevention and treatment of DCM.

## 1 Introduction

The growing number of diabetics is closely linked to the poor lifestyles of modern people. According to the report, the number of people with diabetes will grow from the current 537 million to nearly 800 million by 2045. The development of diabetes is influenced by a number of factors, such as excess weight and body fat due to excessive diet, metabolic disorders due to work stress and lack of sleep, and a family history of diabetes (1–3). However, diabetes is not the main cause of increased mortality and financial stress, but the complications of diabetes, which has become a global health problem. Diabetic cardiomyopathy (DCM), defined as a microvascular complication of diabetes after the exclusion of ventricular diastolic or systolic dysfunction other than hypertension, coronary artery disease and valvular disease, is one of the leading causes of death in diabetic patients (1, 4, 5).

**Figure 1:**
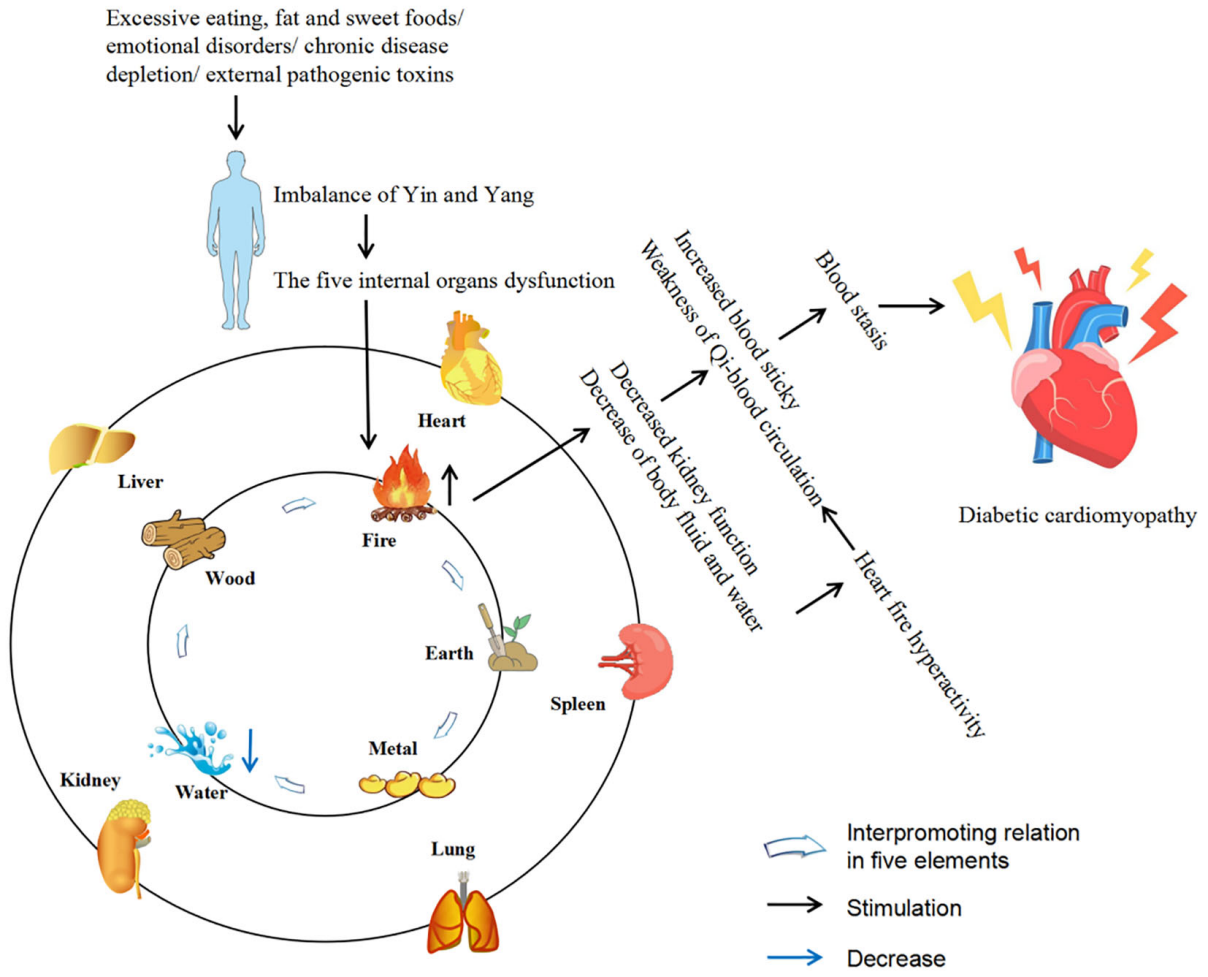
Description of the pathogenesis of DCM from the perspective of TCM. The five internal organs include liver, heart, spleen, lung and kidney. The five elements include wood, fire, earth, metal and water, which are often used to describe the attributes of the five internal organs (liver belongs to wood, heart belongs to fire, spleen belongs to earth, lung belongs to metal, kidney belongs to water).

**Figure 2:**
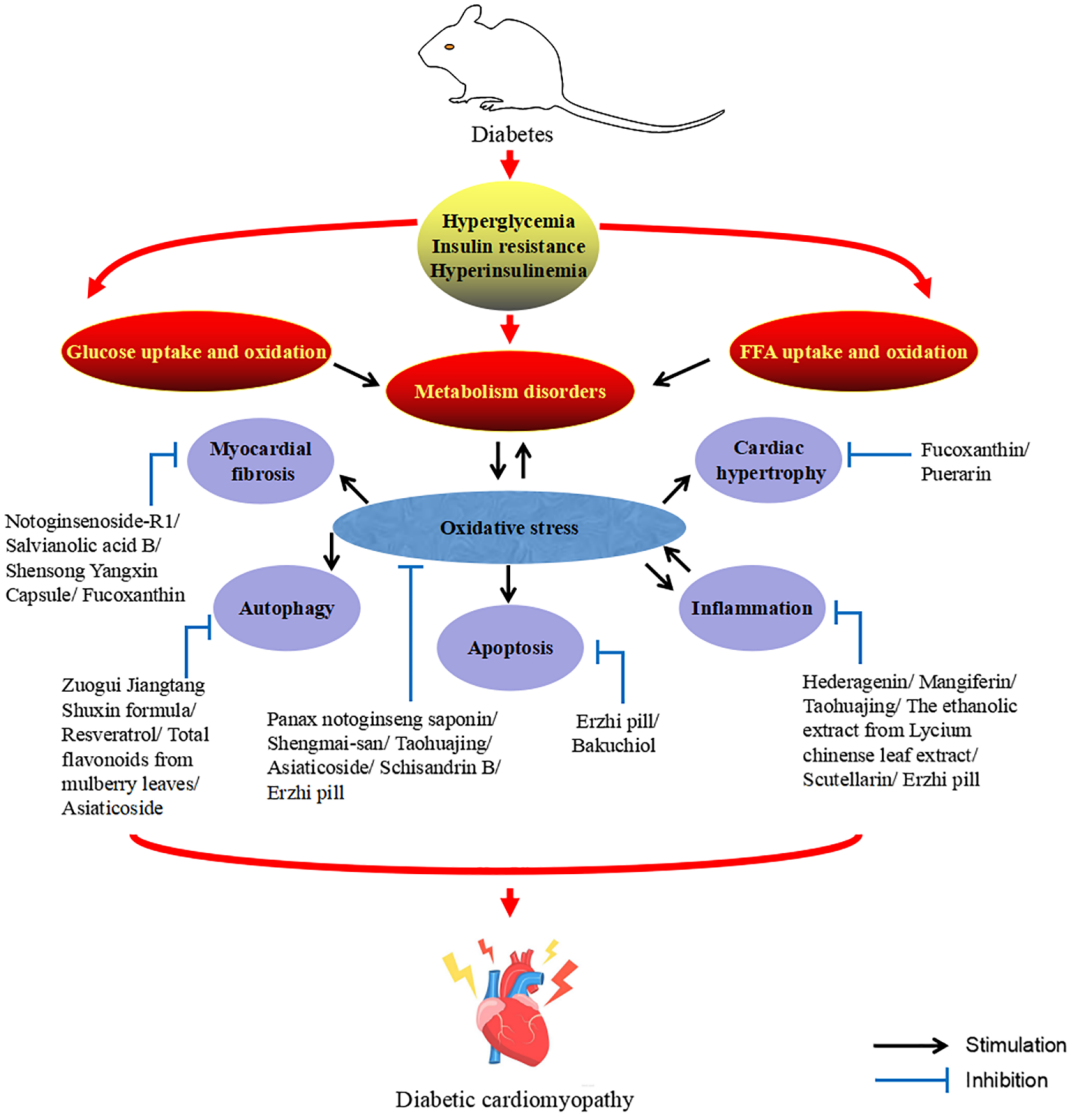
Therapeutic potential of TCM against DCM.

The DCM is characterized by abnormal cardiac structure and function and could be divided into two phases based on the main characteristics of the different periods. The early stages are mainly characterized by dysfunction and left ventricular hypertrophy in the absence of vascular defects, while the middle and late stages are characterized by systolic dysfunction and myocardial fibrosis, which ultimately lead to cardiac insufficiency, heart failure and even death (6, 7). DCM is a complex and diverse pathogenesis of heart disease. Although much effort has been devoted to exploring its mechanisms, its pathogenesis has yet to be elucidated. However, at present, the mainstream view is that the pathogenesis of DCM is mainly related to inflammation, oxidative stress, myocardial fibrosis, apoptosis, autophagy, and the crosstalk effects of these factors (6–10). At present, there are many adverse side effects of conventional DCM drugs in clinical practice, and some newly developed targeted drugs also limit their clinical application due to their high cost (11). Therefore, the search for drugs or complementary and alternative therapies to treat and limit the development of DCM is urgent. Traditional Chinese medicine (TCM) has been practiced for thousands of years in the history of Chinese medicine. With its unique system of diagnosis and treatment, TCM is the crystallization of ancient Chinese wisdom. The theories of Yin and Yang, the five elements, and the five internal organs, which complement each other, form the theoretical basis of TCM. Yin and Yang are in a state of dynamic balance under normal circumstances, when some external factors such as excessive eating, fat and sweet, emotional disorders, chronic disease depletion, external pathogenic toxins or the cascade of the above factors affect the body, the imbalance of Yin and Yang will lead to the dysfunction of the five internal organs, and then cause damage to the body. Before the rise of modern medicine, TCM was the main way for people to prevent and treat diseases. Compared with synthetic drugs with a single target activity, TCM has the natural advantages of safety, multi-component and multi-target, and could more effectively deal with DCM with complex and diverse pathogenesis. Diabetes belongs to the category of “Xiaoke” in TCM, and modern TCM scientists call DCM “Xiaoke heart diseases”. Although there was no name of DCM in ancient literature, according to its clinical manifestations, it was classified as “Xiaoke diseases” complicated with “chest numbness” and “palpitation”. There were also relevant records in literature describing its basic pathogenesis and clinical manifestations, indicating that there had been certain clinical experience in the prevention and treatment of DCM in ancient times (12–15). Excessive dryness and heat inside is the core pathogenesis of Xiaoke disease. Dryness and heat are more dominant, and Qi-blood essence and fluid are consumed, especially the damage of Yin is more severe, so Yin deficiency and dryness-heat are caused, Yin deficiency is the foundation and dryness-heat is the surface. Long onset of disease can cause the decrease of body fluid and water, resulting in the blood sticky and the weak operation of Qi and blood, causing blood stasis. In addition, the heart belongs to the fire viscera, the kidney is the viscera of the water, the kidney water needs the warmth of the heart fire, so that the kidney water is not cold, and the heart fire needs the nourishment of the kidney water to make the heart fire not excessive. The depletion of body fluids leads to heart fire burning the heart’s blood vessels, causing poor circulation of Qi and blood and resulting in blood stasis, which eventually leads to DCM. Qi-blood disharmony is an important pathogenesis of DCM, and it runs through the whole disease process. The weakness of the body, improper diet, and excessive eating, fat and sweet cause the spleen and stomach transport loss, wet turbidness endogenous, Qi machinery block. In addition, emotional disorders can also lead to stagnation of Qi. Qi injury leads to weak blood transport, poor blood flow and blockage of the heart pulse, resulting in DCM (12–14). **Figure 1** describes the pathogenesis of DCM from the perspective of TCM. So far, TCM has accumulated a large amount of clinical experience and research data in the prevention and treatment of DCM. This study summarizes the relevant pathological mechanisms of TCM treatment of DCM in recent years in order to provide new ideas for the clinical use of TCM treatment of DCM. Therapeutic potential of TCM against DCM (**Figure 2**).

## 2 TCM interferes with the inflammation of DCM

It is well known that chronic inflammation is a signature change in the pathophysiological processes of DCM and could mediate early structural and metabolic changes in the heart, such as left ventricular hypertrophy, abnormal calcium processing, systolic impairment, myocardial fibrosis, and apoptosis. Hyperglycemia promotes the release of cytokines and chemokines, which interact to exacerbate the inflammatory response. Tumor necrosis factor-α (TNF-α), interleukin-6 (IL-6), IL-8, IL-1β and C-reactive protein (CRP) are the most representative inflammatory markers. Although it is not clear which one or more causes the myocardial pathological changes, the activation of nuclear factor kappa B (NF-κB) is the same end point, which causes reactive oxygen species (ROS) stress, leading to myocardial remodeling and fibrosis, and the result is myocardial diastolic dysfunction (16, 17).

Hederagenin (HED) is a natural compound of pentacyclic triterpenoid isolated from the leaves of Cyclocarya paliurus and other common plants, which has anti-inflammatory, anti-diabetic and anti-atherogenic biological activities (18, 19). Li et al. (19) found that HED reduced body mass and heart mass and improved cardiac dysfunction in diabetic mice, but had no effect on blood glucose. Results from WGA staining and Masson’s trichrome staining showed that HED improved myocardial hypertrophy and fibrosis in diabetic mice. The plasma levels of pro-inflammatory cytokines TNF-α, IL-1β and IL-6 in diabetic mice were significantly increased, and the expression levels of these pro-inflammatory cytokines could be significantly reduced after the HED intervention. In addition, HED inhibited the nuclear translocation of NF-κB and Smads, and down-regulated the expression levels of transforming growth factor (TGF)-β1 and collagen I. These results suggest that HED could improve DCM by inhibiting the activation of NF-κB and Smads signaling to reduce myocardial inflammatory response.

Mangiferin (MG) is a natural polyphenolic compound derived from *Anemarrhena* rhizome, which has beneficial anti-inflammatory, anti-diabetic, antioxidant and anti-fibrotic activities (20, 21). Hou et al. (21) reported the intervention effect of MG on high-fat diet combined with streptozotocin (STZ)-induced DCM rats. Fasting blood glucose (FBG), CK-MB, LDH, serum total cholesterol (TC) and total triglyceride (TG) levels were significantly increased in DCM rats, and after MG intervention, their increase could be significantly reversed. Masson trichrome staining results showed that MG inhibited collagen aggregation, decreased collagen volume fraction and myocardial fibrosis in DCM rat. In addition, MG decreased the expression levels of advanced glycation end products (AGEs), receptor for advanced glycation end products (RAGEs) and ROS in DCM rats. In the mechanism study, MG could down-regulate the levels of inflammatory factors TNF-α and IL-1β, inhibit the expression level of NF-κB and nuclear translocation of NF-κB in the myocard-tissue of DCM rats, indicating the anti-inflammatory ability of MG. These results demonstrate the potential of MG to improve DCM through anti-inflammatory effects.

Taohuajing (THJ) is a TCM formula used in clinical treatment of DCM, mainly composed of Persicae semen, Polygonatum sibiricum and Carthami flos (22). Yao et al. (22) found that THJ could improve blood glucose, insulin sensitivity, blood lipid and heart function in diabetic mice. Masson and HE staining showed that THJ was able to ameliorate damage to myocardial function such as increased collagen, damage to cardiomyocytes and mast cells, mitochondrial swelling, and myofibril rupture in the hearts of DCM mice. In terms of mechanism studies, THJ decreased the expression levels of TNF-α, IL-6 and IL-1β in DCM mice, indicating its anti-inflammatory effect. In addition, THJ could reduce the levels of ROS and MDA, and increase the levels of SOD and GSHPx, suggesting its ability to against oxidative stress. The results suggest that THJ has the ability to improve DCM, and this ability is related to the anti-inflammatory and antioxidant stress of THJ. *Lycium chinense* is a well-known plant with medicinal and food homology. Its fruit, root and leaf have excellent nutritional and medicinal value, and have anti-inflammatory, anti-obesity and anti-oxidation effects (23, 24). Wen et al. (24) observed the intervention effect of the ethanolic extract from Lycium chinense leaf extract (LCME) on diabetic rats. The diabetic rats had significantly reduced body mass and heart mass, significantly increased FBG, irregular muscle fibers in the cardiac tissue, hypertrophy, and infiltration of inflammatory cells, which were clearly reversed after LCME intervention. In addition, LCME down-regulates the overexpression of inflammatory markers IL-6, IL-1β, TNF-α and NF-κB in the heart tissue of diabetic rats. The results suggest that LCME could improve DCM by alleviating myocardial inflammation. Andrographolide (AG) is a diterpene lactone compound that could be used clinically for the treatment of cardiovascular diseases and diabetes (25, 26). Liang et al. (27) reported the intervention effect of AG on STZ-induced diabetes mice. In diabetic mice, the levels of LVEF, fractional shortening (FS), E/A and insulin were reduced, and the levels of blood glucose were increased. After the intervention of AG, these indices improved significantly, indicating that AG had a beneficial effect on myocardial dysfunction in diabetic mice. In addition, AG also decreased the increase of Collagen deposition in myocardial interstitial region, and the increase of Collagen I, Collagen indigenous, fibronectin (FN), ANP and BNP in diabetic mice. In terms of mechanism research, AG reduces the expression levels of inflammatory mediators intercellular cell adhesion molecule-1 (ICAM-1), vascular cell adhesion molecule-1 (VCAM-1), TNF-α, IL-1β and IL-6 in myocardial tissue of diabetic mice, and down-regulates the overexpression of the key proteins p-IκBα and p65-NF-κB in the NF-κB pathway. Similarly, in the high glucose (HG)-induced H9c2 cardiomyoblasts model, AG also down-regulated the overexpression of p-IκBα and nucleus p65-NF-κB. These *in vitro* and *in vivo* results strongly suggest that AG could improve DCM through anti-inflammatory effects. Scutellarin (SCU) is a flavonoid derived from Erigeron flower, which has antioxidant and anti-inflammatory effects and has protective effects on diabetic complications such as diabetic nephropathy, diabetic retinopathy, diabetic liver injury and diabetic testicular injury (28, 29). Huo et al. (29) found that SCU decreased FBG, TC, TG and low-density lipoprotein (LDL) in diabetic mice, and increased high-density lipoprotein (HDL). It showed the ability to improve blood glucose and lipids in diabetic mice. SCU improved cardiac function in diabetic mice by reducing CK-MB, Troponin and BNP levels. In the results of Masson trichrome staining, diabetic mice have shown cardiomyocyte necrosis, vacuolation and inflammatory cell infiltration with interstitial fibrosis, and these myocardial injuries have been improved after SCU intervention. In further mechanism studies, SCU could down-regulate the expression level of inflammatory markers TLR4, Myd88, NF-κB, IL-6 and TNF-α, and up-regulate the expression level of IκBβ, indicating the anti-inflammatory effect of SCU. These results suggest that SCU could improve DCM, and its mechanism may be related to inhibiting myocardial inflammation.

Moreover, Fufang Zhenzhu Tiaozhi (30), Schisandrin B (31), Syringaresinol (32) and Myricitrin (33) have shown significant effects in alleviating DCM by mechanism related to the inhibition of inflammation.

## 3 TCM interferes with the oxidative stress of DCM

Currently, it is unclear whether the pathogenesis of DCM is directly attributable to metabolic dysfunction or secondary to microvascular disease in diabetes, but it is certain that oxidative stress is one of the important causes of DCM pathogenesis. Oxidative stress refers to the production and elimination of ROS in the cell, the destruction of the dynamical balance between oxidation and antioxidation, and finally the destruction of biological macromolecules. ROS production in cardiomyocytes is a vicious cycle process that could lead not only to further ROS production, but also to mitochondrial DNA damage, lipid peroxidation and protein post-translational modification, and even to inflammation, cardiac hypertrophy and fibrosis, and their interaction could lead to irreversible cell damage, death and cardiac dysfunction (34–36).

Shengmai-san (SMS) is a classic TCM formula for the prevention and treatment of cardiovascular diseases, which has been used in China for thousands of years (37, 38). Lu et al. (38) reported the intervention effect of SMS on STZ-induced diabetic rats. Electron microscopy analysis, Masson’s trichrome and HE staining results showed that the distribution of myocardial collagen fibers and interstitial fibrosis were significantly decreased, myocardial fiber disorders, increased voids, and myocardial fiber collapse, the fibrous region of mitochondria has an uneven, broken and swollen appearance of diabetic rats, which could be significantly improved after SMS intervention. 8-iso-PGF2α and 8-OHdG represent lipid peroxidation and DNA damage states, respectively. SMS could inhibit the increased expression of 8-iso-PGF2α and 8-OHdG in the heart tissue of diabetic rats. T-AOC, which represents the total antioxidant capacity, was significantly reduced in diabetic rat heart tissue, and its decline could be reversed after SMS intervention. These results suggest that SMS could improve the antioxidant capacity of diabetic patients and protect the heart muscle from oxidative damage.NOX is one of the main sources of ROS in the myocardium, and SMS could down-regulate NOX2 and NOX4 in the myocardium of diabetic rats. In addition, the transfer of p47phox and p67phox from the cytoplasm to the cardiac membrane is a prerequisite for NOX activation, and SMS could inhibit the transfer of p47phox and p67phox from the cytoplasm to the cardiac membrane, suggesting that SMS could inhibit NOX activation. It further demonstrated the antioxidant stress ability of SMS. These results suggest that SMS could play a role in the treatment of DCM by ameliorating myocardial damage through anti-oxidative stress.

*Panax notoginseng* saponin (PNS) is the main active ingredient in *Panax notoginseng*, which has the effects of antioxidation, anti apoptosis and anti endoplasmic reticulum stress, and can be used to prevent and treat cardiovascular diseases (39, 40). Zhang et al. (40)found that PNS decreased the levels of TG, TC and LDL and improved the body mass of diabetic mice, but had no significant effect on FBG, suggesting that PNS improved insulin resistance mainly by reducing lipid levels, rather than reducing blood glucose levels. PNS also decreased leptin levels and increased adiponectin levels, and improved lipid accumulation in adiponectin cells in diabetic mice, further confirming the ability of PNS to improve obesity and insulin resistance in diabetic mice. The results of Red O staining showed the presence of a large number of lipid droplets in the hearts of diabetic mice, which reversed upon PNS intervention. This result was also verified *in vitro* with palmitate acid (PA)-cultured H9c2 cells inducing lipotoxicity. In the study of related antioxidant function, PNS increased the expression of superoxide dismutase (SOD), catalase (CAT) and glucose peroxidase (GPx) and decreased the expression of malondialdehyde (MDA) in the serum of diabetes mice, suggesting the antioxidant capacity of PNS. Mitochondria are the main organelles that produce ROS, and PNS was also shown to improve mitochondrial damage in diabetic mice, and therefore myocardial oxidative stress damage in diabetic mice. These results suggest that PNS could improve cardiac function by regulating lipid metabolism, inhibiting oxidative stress and protecting mitochondrial function, and thus play a role in improving DCM. Erzhi pill (EZP) made of *Ligustrum lucidum* W. T. Aiton and *Eclipta prostrata* (L.) L. in equal proportions, it has the effect of nourishing Yin and tonifying liver and kidney. Peng et al. (41) observed the effect of EZP on DCM in diabetic rats induced by high fat diet combined with STZ-induced diabetes. The serum levels of LDL, TG, TC and FBG were significantly increased and HDL was significantly decreased in diabetic rats, and their levels could be reversed after EZP intervention. In addition, EZP could improve the structural disorder of the myocardium, myocardial cell looseness and hypertrophy, and pathological manifestations of striated muscle rupture in DCM rats. In further studies, EZP increased the expression levels of SOD, CAT and GPx, and decreased the expression levels of ROS and MDA in myocardial tissue of diabetic rats. These results suggest that EZP could improve DCM by improving blood glucose and lipids, and inhibiting myocardial oxidative stress.

Moreover, Mulberry granules (42), the total saponins of *Aralia taibaiensis* (43), Schisandrin B (44) and asiaticoside (45) have shown significant effects in alleviating DCM by mechanism related to the inhibition of oxidative stress.

## 4 TCM interferes with the myocardial fibrosis of DCM

Myocardial fibrosis is a hallmark of diabetic cardiomyopathy, an interstitial expansion of the myocardium caused by excessive deposition of extracellular matrix (ECM) proteins. In general, there are two main types of myocardial fibrosis: replacement fibrosis and interstitial fibrosis. The former, commonly associated with myocardial infarction, is characterized by the replacement of dead cardiomyocytes by collagen-rich scar tissue due to ischemia, resulting in replacement fibrosis, and is thought to be a form of self-protection against pathological damage. The latter is mainly caused by the accumulation of ECM proteins and the enlargement of intima and perimuscular space. In addition, interstitial fibrosis is a common pathological feature of the major types of myocardial fibrosis and is associated with a variety of pathological conditions, such as diabetes, heart failure, and hypertension. The pathogenesis of myocardial fibrosis in DCM is unclear, but most researchers believe that it is caused by the cross-pollination of multiple factors, such as persistent hyperglycemia, inflammation, and oxidative stress (46, 47).

*Panax notoginseng (Burk.)* F.H. Chen (PN) is a very popular TCM with various properties and is often used in clinical practice to regulate various cardiovascular diseases. Notoginsenoside (NG)-R1 is one of the main compounds of PN, which has a good role in protecting cardiovascular and cerebral vessels (48, 49). TGF-β-mediated signaling (such as Smad signaling) helps control the expression of collagen, which is closely related to myocardial fibrosis. Zhang et al. (50) found that NG-R1 downregulates ROS, Smad2/3, p-Smad2, Collagen I in AGEs induced H9c2 cells, promotes the expression levels of estrogen receptor (ER) - α and Smurf2, and reduces cell apoptosis and mitochondrial damage. *In vivo*, NG-R1 improved the cardiac function of diabetic mice by decreasing LVIDd and LV mass and increasing LVVd, LVVs, ejection fraction (EF) and FS. In addition, the elevations of LDH, AST and CK-MB in serum and TGF-β, Collagen I and Smad2/3 in myocardial tissue of diabetic mice were also inhibited by NG-R1. These results suggest that NG-R1 may play a role in the treatment of DCM by ameliorating myocardial injury through anti-myocardial fibrosis.

Salvianolic acid B (SaB) is a water-soluble component of phenolic acids with the largest content in *Salvia miltiorrhiza Bunge*, which has anti-inflammatory, antioxidant, anti-fibrosis and metabolic regulation effects on various organs of human body. It is widely used in the prevention and treatment of cardiovascular and cerebrovascular diseases (51). Li et al. (52) established STZ-induced C57BL/6J diabetes mice model and human umbilical vein endothelial cell model under hypoxia to observe the intervention effect of SaB on DCM. *In vivo*, the heart weight, the ratio of heart weight to body weight, the cross-sectional area and the diameter of cardiomyocytes were significantly increased in diabetic mice, and SaB could reverse their increase. These results indicate that SaB could reverse myocardial remodeling in diabetic mice. The cardiac function indexes LVEDD, LVEF, FS, E/A and E’/A’ of diabetic mice were damaged to different degrees, but after SaB intervention, all of them were improved except LVEDD, which reflects the beneficial effect of SaB on cardiac function injury. In the experiment of Masson trichrome and Sirius Red staying of heart tissue sections, ECM deposition was more in perivascular and intramyocardial regions, and fibrosis markers collagen I and collagen III were increased of diabetic mice, SaB could reverse this diabetes-induced ECM deposition and increase in fibrosis markers. The effect of SaB on reducing fibrosis markers was also confirmed in further immunohistochemical staining. Cardiac staining showed that hyperglycemia reduced blood vessel formation in diabetic mice and thus reduced capillary density, and SaB intervention was able to increase its density, suggesting that SaB has a role in promoting angiogenesis. ​In addition, the pro-angiogenic effect of SaB has been further demonstrated by *in vitro* experiments. Under hypoxia conditions, SaB stimulates the formation of cell tubules and reverses the decrease of VEGFA and VEGFR2, the signature indicators of angiogenesis in the heart caused by hyperglycemia. These evidences strongly support the angiogenic ability of SaB. These results suggest that SaB may play a role in the prevention and treatment of DCM by promoting angiogenesis to improve cardiac fibrosis and myocardial function.

Shensong Yangxin Capsule (SYC) is a classic TCM formula, which has a beneficial effect on the treatment of arrhythmia in clinic (53, 54). Shen et al. (55) observed the intervention effect of SYC on DCM by establishing STZ induced diabetic rats. SYC could improve heart function and reduce heart weight/body weight ratio by upregating EF and FS in diabetic rats, but has no significant effect on abnormal blood glucose and lipid changes. Masson trichrome and HE staining showed significant increase in interstitial fibrosis area, myotome disturbance, interstitial collagen increase and cardiomyocyte hypertrophy in diabetic rats, and SYC intervention could improve the above indicators. In addition, further studies found that SYC down-regulated the expression levels of Collagen I, Collagen III, TGF-β1 and p-Smad2/3 and up-regulated the expression levels of Smad7 in myocardial tissue of diabetic rats. These experimental evidence suggest that SYC may prevent and treat DCM, and that its mechanism may be related to the improvement of myocardial fibrosis.

Moreover, Shengmai San (56), Yi-qi-huo-xue formula (57), artemisinin combined with allicin (58) and triptolide (59) have shown significant effects in alleviating DCM by mechanism related to the inhibition of myocardial fibrosis.

## 5 TCM interferes with the autophagy of DCM

Autophagy, as a regular eukaryotic homeostatic regulation mechanism, is highly conserved in the development of organisms. It is a catabolic process that maintains cellular homeostasis (including cardiomyocytes, endothelial cells and arterial smooth muscle cells) through lysosomal dependent removal of accumulated proteins and damaged organelle components in cardiovascular cells, thus balancing macromolecular biosynthesis and catabolism to protect organisms from cardiovascular diseases. Persistent ROS could cause inflammatory cell infiltration, cardiomyocyte necrosis and myocardial dysfunction, leading to autophagy and thus promoting the process of cell death, so oxidative stress is considered an important intracellular signal transducer to maintain autophagy. In addition to oxidative stress, hyperglycemia, insulin resistance and other factors also have important effects on the process of autophagy (60–65).

Resveratrol (RSV) is a polyphenolic phytoalexin, found in many plants, with antioxidant, anti-inflammatory, anti-diabetic, and heart protective biological activities. It has been reported that RSV has therapeutic effects on cardiovascular diseases such as heart failure, myocardial ischemia and atherosclerosis (66, 67). Ma et al. (65) used C57BL/KSJ db/db mice as a diabetes model to observe the intervention effect of RSV on DCM. Diabetic mice had significantly increased levels of blood glucose, CK, and LDH, while RSV significantly reduced their levels. In diabetic rats, the arrangement of cardiomyocytes is significantly disordered, enlarged, focal necrosis is accompanied by significant inflammatory cell infiltration, and the apoptotic rate of cardiomyocytes is significantly increased. After RSV intervention, the above indexes were significantly improved. Transmission electron microscopy results showed that myocardial cells of diabetic mice became disordered as the cells swelled and the number of autophagosomes decreased significantly. After RSV intervention, the cell arrangement was significantly improved and the number of autophagosomes was significantly increased. In addition, the expression level of autophagy protein p62 in myocardial tissue of diabetic mice was significantly increased, the ratio level of LC3II/LC3I was significantly decreased, and the ratio of apoptotic protein Bax and Bax/Bcl-2 was significantly increased, and the expression level of Bcl-2 was significantly decreased. RSV could down-regulate the levels of p62, Bax and Bax/Bcl-2 and up-regulate the ratio of LC3II/LC3I and the level of Bcl-2. These results suggest that RSV may play a role in the treatment of DCM by up-regulating autophagy levels and inhibiting apoptosis in cardiomyocytes.

Zuogui Jiangtang Shuxin formula(ZJSF) is a traditional TCM formula, which has the effects of nourishing Yin and Qi, promoting blood circulation and detoxification, and is often used in clinical treatment of cardiovascular complications of diabetes (68, 69). Tian et al. (70) established high fat combined with STZ-induced diabetic transgenic MKR mice to observe the intervention effect of ZJSF on DCM. The levels of FBG, cardiac troponin I (cTnI), CK-MB, collagen I and collagen III were elevated in diabetic mice, while ZJSF reduced their elevations. The reversal of these indicators suggests that ZJSF could improve myocardial damage and myocardial fibrosis in diabetic mice. Transmission electron microscopy was used to observe morphological and structural changes in myocardium and autophagosomes. The results showed that the myocardial cells of diabetic mice were disordered, incomplete in structure, stage degeneration of some muscle fibers, significant expansion of intercalated discs, severe swelling of mitochondria, and severe stenosis or even obstruction of microvascular lumen. After the intervention of ZJSF, the microvascular lumen stenosis of cardiomyocytes in diabetic mice was improved, the arrangement of cardiomyocytes and intercalated discs became more orderly, the light and dark bands were clear, the mitochondria became slightly swollen, and the morphological structure of myocardium was improved. In addition, Beclin-1, a marker protein of the myocardial autophagosome membrane, was significantly weakened in diabetic mice, and no autophagosomes were observed. After the intervention of ZJSF, a certain number of bilayer-membrane-structured autophagosomes were observed and the expression of Beclin-1 protein was increased, suggesting that the level of autophagy was up-regulated. These results suggest that ZJSF may play a role in the treatment of DCM by reducing myocardial fibrosis and improving myocardial damage in diabetic mice by up-regulating autophagy levels.

Total flavonoids from mulberry leaves (MLF) is one of the main active components of mulberry leaves, which has beneficial antioxidant, anti-inflammatory and antibacterial potential and is especially known for its excellent antioxidant activity (71, 72). Yang et al. (73) observed the intervention effect of MLF on DCM by establishing STZ-induced diabetes rats. In diabetic rats, CK, LDH were significantly increased and cardiac function indexes LVEF, E/A and IVRT were significantly decreased, while MLF could reverse their levels. MLF decreased the expression levels of inflammatory cytokines IL-1β and TNF-α, and down-regulated the levels of key inflammasome proteins NLRP3, ASC and Caspase-1 in the myocardium of diabetic rats, which indicated the anti-inflammatory ability of MLF. In addition, MLF increased the expression levels of autophagy proteins Beclin-1 and LC3-II in myocardial tissue of diabetic rats, suggesting that MLF could promote autophagy. These results suggest that MLF could play a beneficial role in the development of DCM by inhibiting the activation of the NLRP3 inflammasome in cardiomyocytes, and the mechanism may be related to the induction of autophagy in cardiomyocytes. Moreover, asiaticoside (74) and pterostilbene (75) have shown significant effects in alleviating DCM by mechanism related to the promotion of autophagy.

## 6 TCM interferes with other mechanisms of DCM

Astragalus membranaceus is one of the main members of many anti-diabetic prescriptions and has beneficial effects on diabetic complications. Astragalus polysaccharides (AP), one of the main active ingredients extracted from Astragalus membranaceus, has antioxidant and anti-diabetic biological activities (76). Sun et al. (76) found that STZ-induced diabetic rats’ LVEDD, LVESD, LVEDV and LVESV all increased, and LVFS and LVEF significantly decreased. After AP intervention, the changes of these indexes were reversed. The results suggest that AP improves cardiac function in DCM rats. HE staining results showed that myocardial fiber arrangement was disordered, nucleus size was different, vacuoles increased, and muscle fiber collapse in DCM group, and the intervention of AP could improve these pathological changes in the myocarocytes of DCM rats. Activation of CHOP could induce apoptosis of DCM cells, and CHOP is also a key downstream factor in endoplasmic reticulum stress, which could be regulated by P-PERK and ATF6. The expression levels of CHOP, P-PERK and ATF6 were significantly increased in the cardiomyocytes of DCM rats, but their expression levels were down-regulated after AP intervention. In addition, the percentage of apoptosis of cardiomyocytes in DCM rats was significantly increased by TUNEL staining, while AP could significantly decrease the apoptotic percentage of cardiomyocytes. The above anti-apoptotic ability of AP was further verified in HG-treated H9C2 cells, suggesting that AP could improve DCM by alleviating apoptosis of cardiomyocytes.

The anti-oxidative stress effect of EZP has been mentioned above (41). In addition to the anti-oxidative stress effect, EZP could also reduce the expression levels of inflammatory factors TNF-α, IL-1β and IL-6 in serum of DCM rats. In addition, activation of caspase-3, caspase-8, and caspase-9, as well as levels of Bcl-2 and Bax, are thought to reflect apoptotic activity. The number of TUNEL positive cells in the myocardium of DCM rats increased significantly, the expression levels of caspase-3, caspase-8, caspaes-9 and Bax increased, and the expression levels of Bcl decreased, and their levels could be restored after EZP intervention, suggesting the anti-apoptotic ability of EZP. These results suggest that EZP could also improve DCM through anti-inflammatory and anti-apoptotic effects. Bakuchiol is one of the main active ingredients of Psoralea corylifolia, which has antioxidant, anti-inflammatory and anticancer pharmacological properties (77). Kang et al. (78) established a STZ-induced diabetic rat model to observe the intervention effect of bakuchiol on DCM. In diabetic rats, LVEF was significantly increased, myocardial arrangement was disorganized and perivascular collagen fibers were significantly increased, and the intervention of bakuchiol could ameliorate these changes. In addition, the expression level of Bcl-2 was decreased, and the expression levels of cardiomyocyte apoptosis index, Cleaved caspase-3/caspase-3 and Bax were increased in diabetic rats, and bakuchiol could also reverse their expression levels. It was suggested that bakuchiol could alleviate DCM by improving cardiomyocyte apoptosis.

Fucoxanthin (FT) is a carotenoid derived from natural Marine organisms, accounting for about 10% of carotenoids. FT has a variety of beneficial effects, including anti-inflammatory, antioxidant, anti-obesity and anti-diabetic properties (79, 80). Zheng et al. (80) observed the effect of FT on DCM by establishing STZ-induced diabetic rats and HG-induced H9c2 cells. *In vivo*, HE staining showed hypertrophy and disordered intercellular arrangement of cardiomyocytes in diabetic rats, while FT could improve these phenomena and reduce the cross-sectional area of cells. In addition, FT reduced the elevated expression level of the fibrosis marker protein TGF-β1 in the heart tissue of diabetic rats. *In vitro*, HG-induced H9C2 cardiomyocytes increased surface area, while FT reduced cell surface area and down-regulated mRNA levels of cell mast factors ANP, BNP and β-MHC. The results suggest that FX has a beneficial effect on DCM, and that its mechanism may be related to the inhibition of myocardial fibrosis and myocardial hypertrophy. Similarly, puerarin (81) also showed inhibition of myocardial hypertrophy on HG-induced H9C2 cells, indicating puerarin’s anti-DCM potential.

## 7 Discussion

TCM has occupied an important position in the history of Eastern medicine in the past several thousand years, and is the core means of preventing and treating diseases. It is a traditional discipline with a completely different system from Western medicine, with a complete theoretical system and extensive clinical practice history. The biggest difference lies in its holistic view of treatment, where the spread and variability of the disease will be taken into account along with symptomatic treatment. Given the complexity of DCM, a holistic approach may be a more appropriate approach. In recent years, people’s interest in TCM has reached an unprecedented height, because it not only has the advantages of low price, few side effects and good efficacy, but also can be customized according to the patient’s disease changes, which has obvious benefits for both the treatment effect of TCM and the cumulative toxicity of long-term use of the same TCM. Thus TCM, whether used alone or in combination with Western medicine, is a potential complementary and alternative treatment for DCM.

This review describes the therapeutic potential of TCM against DCM from the perspective of pathological mechanism (**Table 1**). Studies have found that the mechanism of TCM against DCM is mainly related to inflammation, oxidative stress, myocardial fibrosis, autophagy and apoptosis, and multiple factors usually play a synergistic role. In this study, HED, MG, THJ, LCME, AG and SCU could inhibit inflammation, SMS, PNS and EZP could inhibit oxidative stress, NG-R1, SaB and SYC could inhibit myocardial fibrosis, and RSV, ZJSF and MLF improve DCM by promoting autophagy. In addition, many TCMs improve DCM not only by interfering with one pathological process, but multiple, for example, THJ could improve DCM by anti-inflammatory and antioxidant stress; RSV improves DCM by promoting autophagy and inhibiting apoptosis; ZJSF improves DCM by promoting autophagy and inhibiting myocardial fibrosis; MLF improves DCM by promoting autophagy and anti-inflammatory; FX improves DCM by inhibiting myocardial fibrosis and hypertrophy; EZP improves DCM by anti-oxidative stress, anti-inflammation and anti-apoptosis. These studies reflect the multi-level and multi-target nature of TCM. Considering the complexity and long-term nature of DCM, TCM with multiple levels, multiple targets and few side effects has great potential as a candidate drug for the treatment of DCM.

**Table 1:** The experimental evidence of TCM in the treatment of DCM.

Mechanisms	Agents	Experiment models		Outcomes of TCM interventions	References
		*In vivo*	*In vitro*		
Anti-inflammation	Hederagenin (HED)	db/db mice	–	Improve myocardial hypertrophy and fibrosis, and decrease expression of inflammatory markers	Li et al. (19)
	Taohuajing (THJ)	C57BL/6 mice	–	Improve cardiac function and decrease expression of inflammatory factors	Yao et al. (22)
	The ethanolic extract from Lycium chinense leaf extract (LCME)	SD rats	–	Inhibit myocardial damage and decrease expression of inflammatory markers	Wen et al. (24)
	Andrographolide (AG)	C57/BL6J mice	H9c2 cardiomyocytes	Improve myocardial dysfunction and decrease expression of inflammatory markers	Liang et al. (27)
Anti-oxidative stress	Shengmai-san (SMS)	SD rats	–	Inhibit myocardial damage and decrease expression of NOX2, NOX4 and T-AOC	Lu et al. (38)
	Panax notoginseng saponin (PNS)	db/db mice	H9c2 cardiomyocytes	Improve lipid droplets in heart, and decrease expression of MDA increase expression of SOD, CAT and GPx	Zhang et al. (40)
	Erzhi pill (EZP)	SD rats	–	Improve blood glucose and lipids, and decrease expression of ROS, MDA and increase expression of SOD, CAT and GPx	Peng et al. (41)
Anti-myocardial fibrosis	Notoginsenoside-R1 (NG-R1)	db/db mice	H9c2 cardiomyocytes	Improve cardiac function, decrease expression of TGF-β, ROS, Smad2/3, p-Smad2, Collagen I and increase expression of ER-α, Smurf2	Zhang et al. (50)
	Salvianolic acid B (SaB)	C57BL/6J mice	HUVECs	Improve cardiac function and myocardial remodeling, decrease expression of Collagen I and Collagen III and promote angiogenesis	Li et al. (52)
	Shensong Yangxin Capsule (SYC)	Wistar rats	–	Improve cardiac function, decrease expression of Collagen I, Collagen III, TGF-β1 and p-Smad2/3, and increase expression of Smad7	Shen et al. (55)
Regulating-autophagy	Resveratrol (RSV)	C57BL/KSJ db/db mice	–	Decrease expression of p62, Bax and Bax/Bcl-2, increase expression of the ratio of LC3II/LC3I, Bcl-2 and the number of autophagosomes	Ma et al. (65)
	Zuogui Jiangtang Shuxin formula (ZJSF)	MKR mice	–	Inhibit myocardial damage, decrease expression of CK-MB, collagen I and collagen Ш and increase expression of Beclin-1	Tian et al. (70)

TCM shows a broad prospect in the prevention and treatment of DCM, but there are some limitations. First, the difference of TCM ingredients. Regional differences lead to different growth environments (including climate and soil and other factors), resulting in different TCM components, which directly affect the curative effect. Therefore, it is recommended to conduct fingerprint profiling analysis and standardized techniques such as good manufacturing practices for TCM formulations, so as to reduce the therapeutic effect differences caused by the differentiation of TCM components. Second, there is a lack of large clinical trials. Although TCM has been shown to work in many animal and cell models, will it have similar positive effects when applied to the clinic? That is not yet clear. In addition, the safety of TCM also deserves attention. Therefore, its efficacy and safety can be evaluated by increasing pilot studies and clinical trials. Third, differences in TCM doctor’ syndrome differentiation standards. Much of the knowledge about TCM syndromes is derived from school teaching and empirical inheritance, and there are many schools of TCM, so differences in how TCM doctors differentiate syndromes in the same patient will result in differences in the final treatment effect. Furthermore, the dialectical criterion is also the primary obstacle hindering the use of TCM in Western medicine. If the syndrome types can be determined through integrating omics technologies to validate TCM mechanisms and artificial intelligence-assisted diagnosis, this will not only standardize the diagnostic criteria to the greatest extent possible, but also make TCM easy to use in Western medicine. Four, there is a lack of comparative studies between TCM therapies and conventional DCM therapies, such as differences in their efficacy, cost and side effects. Furthermore, in most preclinical studies of TCM against DCM, there is also a lack of strict positive and negative controls. It is suggested to carry out research in these aspects so as to have a better understanding of the similarities and differences between them, in order to better treat DCM. Five, most of the TCM formulas are hospital preparations, and the production process standards are not clear and uniform. Finally, it is worth noting that for each of the above-mentioned studies on TCM against DCM, there are still problems such as small sample size and lack of blinding, and the quality of the included studies needs to be evaluated. Therefore, definition A: The experimental model includes animal and cell models, and there are positive or negative control groups. B: The experimental models include animal and cell models, but there are no positive or negative control groups. C: The experimental model only includes animal models. D: The experimental model only includes the cell model. The study of quality grade A not only draws relevant conclusions from *in vivo* experiments, but can be further validated by *in vitro* experiments, as well as positive or negative control groups, to confirm the reliability of the results. This is a relatively high level of experimental evidence. Research with a quality level of B is second only to A and also belongs to a relatively high level of experimental evidence. Studies with quality grades C and D belong to slightly lower levels of evidence compared to A and B, especially grade D. Due to the singularity of the cells themselves and their growth environment, their complexity is far lower than that of animals. The evidence that often appears in cell experiments may not necessarily be effectively verified in animal experiments. Therefore, level D is considered as low-quality evidence in these studies. In conclusion, all evidence needs to be viewed dialectically. In general, TCM needs to establish a unified and convincing set of standards guided by the fundamental theories of TCM, and combine with modern medicine to advance the TCM treatment of DCM while giving full play to its advantages.

In conclusion, although TCM still has some limitations, it has great potential to treat DCM through inflammation, oxidative stress, myocardial fibrosis, autophagy and apoptosis. With future in-depth studies on TCM against DCM, new against DCM candidate drugs or complementary and alternative therapies should be screened to provide more ideas and evidence for the clinical use of TCM to prevent and treat DCM.
